# tmRNA Is Essential in *Shigella flexneri*

**DOI:** 10.1371/journal.pone.0057537

**Published:** 2013-02-25

**Authors:** Nitya S. Ramadoss, Xin Zhou, Kenneth C. Keiler

**Affiliations:** Pennsylvania State University, Department of Biochemistry & Molecular Biology, University Park, Pennsylvania, United States of America; Baylor College of Medicine, United States of America

## Abstract

Nonstop mRNAs pose a challenge for bacteria, because translation cannot terminate efficiently without a stop codon. The *trans*-translation pathway resolves nonstop translation complexes by removing the nonstop mRNA, the incomplete protein, and the stalled ribosome. P1 co-transduction experiments demonstrated that tmRNA, a key component of the *trans*-translation pathway, is essential for viability in *Shigella flexneri*. tmRNA was previously shown to be dispensable in the closely related species *Escherichia coli*, because *E. coli* contains a backup system for *trans*-translation mediated by the alternative release factor ArfA. Genome sequence analysis showed that *S. flexneri* does not have a gene encoding ArfA. *E. coli* ArfA could suppress the requirement for tmRNA in *S. flexneri*, indicating that tmRNA is essential in *S. flexneri* because there is no functional backup system. These data suggest that resolution of nonstop translation complexes is required for most bacteria.

## Introduction

mRNAs that lack a stop codon can originate from many events, including premature transcription termination, physical or chemical damage to a complete mRNA, or nucleolytic activity. Translation of a nonstop mRNA is problematic, because termination requires a stop codon. Release factors specifically recognize a stop codon in the ribosomal A site and promote hydrolysis of the peptidyl-tRNA, releasing the newly-synthesized protein and the ribosome [1], [2]. Eukaryotes have mRNA proofreading mechanisms to limit translation initiation on nonstop mRNAs [3]. However, bacteria lack most mRNA proofreading mechanisms and ribosomes frequently translate to the end of a nonstop mRNA [4], generating a nonstop translation complex composed of a truncated mRNA, an incomplete nascent polypeptide, and a ribosome that cannot elongate or terminate translation by the canonical reactions. These nonstop translation complexes are resolved by *trans*-translation, a reaction mediated by tmRNA and a small protein, SmpB [5]. tmRNA contains a tRNA-like acceptor stem and a reading frame encoding a short peptide. SmpB binds tmRNA with high affinity [6]. During *trans*-translation, tmRNA-SmpB recognizes the nonstop translation complex and promotes translation of the tmRNA-encoded peptide onto the end of the nascent polypeptide [7], [8]. This reaction releases the ribosome at a stop codon within tmRNA. The tmRNA-encoded peptide is recognized by several proteases, so the incomplete protein is rapidly degraded [9], [10], [11], [12]. *trans*-Translation also stimulates degradation of the nonstop mRNA, so all components of the nonstop translation complex are efficiently removed [13], [14].

*trans*-Translation occurs with high frequency in bacteria, and is found throughout the bacterial kingdom. Estimates from *E. coli* suggest that 2–4% of translation reactions end in *trans*-translation [4]. Genes encoding tmRNA (*ssrA*) and SmpB (*smpB*) have been identified in all sequenced bacterial genomes, indicating that *trans*-translation confers a selective advantage in all environments that can support bacterial life [15].

The abundance and ubiquity of *trans*-translation suggest that it is very important, and in some species *ssrA* and *smpB* are essential [16], [17], [18], [19]. However, mutants of *E. coli K12* lacking *trans*-translation activity are viable and have only mild growth defects in typical culture conditions [20], [21]. A screen for *E. coli* genes that cannot be deleted in Δ*ssrA* cells identified *arfA*, which encodes an alternative release factor [22]. ArfA binds nonstop translation complexes and recruits RF-2 to hydrolyze the peptidyl-tRNA, releasing the nascent polypeptide and ribosome [23], [24]. ArfA is a backup system for *trans*-translation, because it is only produced when *trans*-translation activity is limiting [25], [26]. In *E. coli*, *arfA* mRNA contains an RNase III cleavage site 5′ of the stop codon, so expression of *arfA* will result in a nonstop complex [25]. When *trans*-translation is functional, ArfA will be tagged and degraded. However, if *trans*-translation is limiting, the truncated but active ArfA will be released [25]. *arfA* genes have been found in the genome sequences of many bacteria, including most enteric gamma-proteobacteria [22], [26].

In this paper we show that *ssrA* is essential in *S. flexneri*, a human pathogen that causes acute dysentery. *S. flexneri* is closely related to *E. coli* [27]. In fact, the *Shigella* and *Escherichia* genera are phylogenetically indistinguishable [27]. *S. flexneri* lacks *arfA*, but when *E. coli arfA* is expressed in *S. flexneri, ssrA* can be deleted. These results suggest that *trans*-translation is essential in *S. flexneri* because it is the only available mechanism to resolve nonstop translation complexes.

## Results and Discussion

### *ssrA* is essential in *S. flexneri*

**Figure 1:**
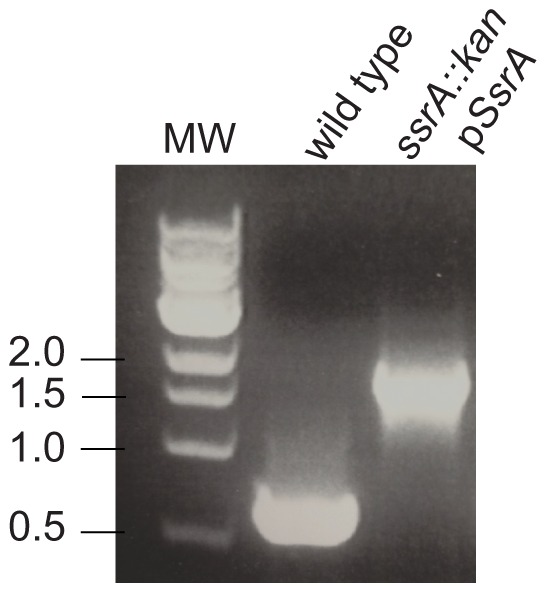
*ssrA* is dispensible in *S. flexneri* when a second copy of the gene is provided. Diagnostic PCR reactions were used to verify deletion of *ssrA* in *S. flexneri ssrA::kan pSsrA*. The expected product size for wild-type *ssrA* is 0.6 kb and for *ssrA::kan* is 1.7 kb. A control reaction using genomic DNA from wild-type *S. flexneri* and molecular weight markers with sizes in kb are indicated.

Efforts to replace *ssrA* in the chromosome of *S. flexneri 2a 2457T* with a kanamycin-resistance gene using Red-mediated recombination were not successful in wild-type cells. However, when a second copy of *ssrA* was provided on a plasmid (pSsrA), kanamycin-resistant colonies were recovered. Diagnostic PCR reactions confirmed that *ssrA* was deleted in kanamycin-resistant cells containing pSsrA (Fig. 1). These results suggested that *ssrA* is essential in *S. flexneri*.

The requirement for *ssrA* was confirmed using a co-transduction experiment. A marker linked to the chromosomal *ssrA* locus was introduced into *S. flexneri* by transducing *zfg-2003::Tn10* from a donor *E. coli* strain. The Tn10 insertion in *zfg-2003* confers tetracycline resistance, and is located ∼0.25 minutes from *ssrA*. *S. flexneri zfg-2003::Tn10* was then transformed with pSsrA and the chromosomal copy of *ssrA* was replaced with a kanamycin-resistance gene to produce *S. flexneri zfg-2003::Tn10 ssrA::kan pSsrA*. P1 lysates were prepared from this strain and used to measure co-transduction of *ssrA::kan* and *zfg-2003::Tn10* into *S. flexneri* strains. The co-transduction frequencies were measured by selecting for tetracycline-resistant transductants and screening these transductants for kanamycin resistance. Based on the map distance between *zfg-2003* and *ssrA*, the tetracycline-resistance gene and kanamycin-resistance gene should be co-transduced with a frequency of ∼70% if *ssrA* were not essential. When *S. flexneri pSsrA* was used as a recipient, the co-transduction frequency was 72±4%, close to the theoretical value. However, when wild-type *S. flexneri* with no additional copy of *ssrA* was used as a recipient, no kanamycin-resistant colonies were recovered from 550 tetracycline-resistant transductants. If *ssrA* were not essential, the probability of obtaining no co-transductants in these experiments would be (0.3)^550^, or ∼10^−288^. These results show that unlike *E. coli K12*, *S. flexneri* requires *ssrA* for viability.

### *S. flexneri* strains do not have *arfA*

**Figure 2:**
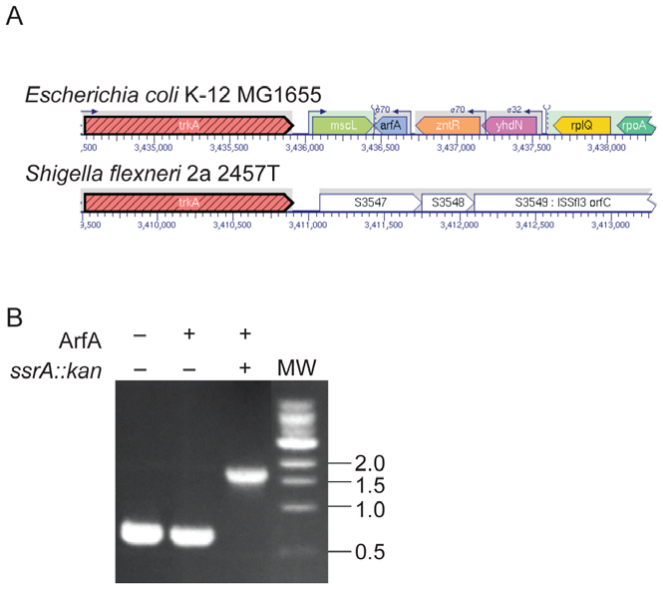
*arfA* accounts for phenotypic differences produced by deleting *ssrA* in *E. coli* and *S. flexneri.* (**A**) *arfA* (blue) in *Escherichia coli K-12 MG1655* and the corresponding locus in *Shigella flexneri* 2a 2457T, aligned using EcoCyc Pathway Tools (SRI International). (**B**) Diagnostic PCR reactions of genomic DNA prepared from wild-type *S. flexneri* (lane 1), *S. flexneri pCA24N-His_6_-ArfA* (lane 2), and *S. flexneri ssrA::kan pCA24N-His_6_-ArfA* (lane 3). The expected product size for wild-type *ssrA* is 0.6 kb and for *ssrA::kan* is 1.7 kb. Molecular weight markers with sizes in kb are indicated.

*E. coli* can survive without *trans*-translation activity because *arfA* is expressed in the absence of *trans*-translation and ArfA activity can resolve nonstop translation complexes [22], [23], [24], [25]. Deletions of *ssrA* and *arfA* in *E. coli* are synthetically lethal [22]. Searches of genome sequences of *Shigella* species using BLAST [28] revealed that *arfA* homologs are present in *S. boydii*, *S. sonnei*, and *S. dysenteriae*, but not in *S. flexneri*. In *Escherichia* and *Shigella* species that have *arfA*, the gene is encoded between *mscL* and *zntR*, 3′ of *trkA*. This chromosomal locus in *S. flexneri* strains contains an insertion element 3′ of *trkA*, suggesting that *arfA* has been deleted by genetic rearrangement (Fig. 2A).

### *E. coli* ArfA can suppress the lethal phenotype of *ssrA* deletion in *S. flexneri*

Given the close phylogenetic relationship between *E. coli* and *S. flexneri*, it was surprising that the phenotypes caused by deleting *ssrA* were so different. However, the absence of *arfA* in *S. flexneri* suggested that the difference might be due to the absence of a backup mechanism for *trans-*translation in *S. flexneri*. To determine if *E. coli* ArfA could suppress the requirement for *ssrA* in *S. flexneri*, a plasmid encoding His6-ArfA from the ASKA collection (pCA24N-His_6_-ArfA) was transformed into *S. flexneri* and these cells were used as the recipient in a co-transduction experiment with P1 lysates from *S. flexneri zfg-2003::Tn10 ssrA::kan pSsrA*. *ssrA::kan* and *zfg-2003::Tn10* were co-transduced into *S. flexneri pCA24N-His_6_-ArfA* with a frequency of 69±8%, indicating that *ssrA* is not essential in cells with pCA24N-His_6_-ArfA. Diagnostic PCR reactions confirmed that *ssrA* was deleted in the kanamycin-resistant cells (Fig. 2B). These results indicated that ArfA can suppress the requirement for *ssrA* in *S. flexneri*.

**Figure 3:**
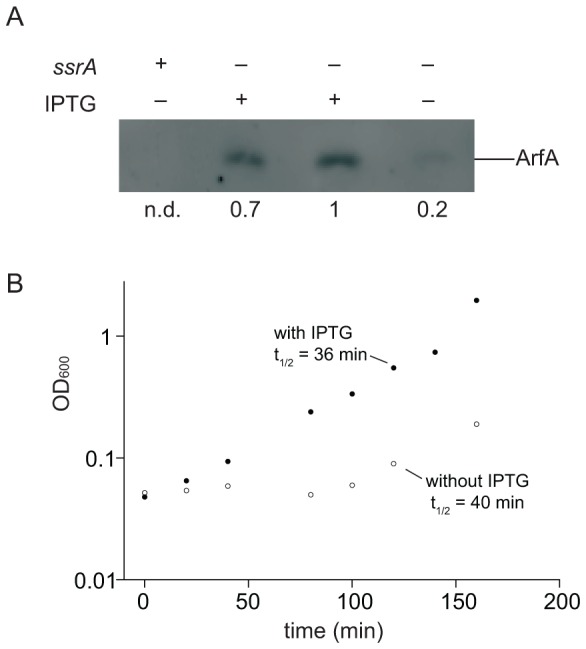
ArfA is expressed in cells containing *pCA24N-His_6_-ArfA*. (**A**) Western blots to determine the expression of ArfA in wild-type *S. flexneri* (lane 1), *S. flexneri ssrA::kan pCA24N-His_6_-ArfA* grown with IPTG at all times (lane 2), grown without IPTG and diluted into medium containing IPTG (lane 3), and grown without exposure to IPTG (lane 4). The amounts of ArfA relative to lane 3 are shown (n.d.: not detectable). (**B**) Growth of *S. flexneri ssrA::kan pCA24N-His_6_-ArfA* with IPTG (closed circles) and with no IPTG (open circles) monitored by optical density at 600 nm. Doubling times during exponential growth (80–160 min) are indicated.

*ssrA* could be deleted in *S. flexneri pCA24N-His_6_-ArfA* cells even when IPTG was not added to induce *arfA* expression. Western blotting revealed that amount of ArfA in uninduced *S. flexneri ssrA::kan pCA24N-His_6_-ArfA* was 15–20% the amount in cells that had been induced (Fig. 3A). When *S. flexneri ssrA::kan pCA24N-His_6_-ArfA* cells were inoculated into fresh medium containing IPTG the lag phase of growth was shorter than when no IPTG was added, but the doubling time during logarithmic growth was similar (Fig. 3B). These data indicate that the amount of ArfA produced in uninduced cultures is sufficient for viability of *S. flexneri* in the absence of *trans*-translation, but higher levels of ArfA are required for optimal growth in culture.

The results described here suggest that all species of the *Escherichia/Shigella* lineage require a mechanism to resolve nonstop translation complexes. For most species in this group *ssrA* is not essential because ArfA acts as a backup system, but because *S. flexneri* does not have *arfA*, *ssrA* is essential. In other proteobacteria, such as *Caulobacter crescentus*, *ssrA* is not essential [29], but there is no ArfA homolog. Perhaps nonstop translation complexes are not as severe a challenge in these species. Alternatively, these species may have a distinct mechanism for releasing nonstop translation complexes.

Like *E. coli, S. flexneri* has a gene encoding ArfB (YaeJ), a second alternative release factor. Purified ArfB can release nonstop translation complexes in vitro, and multicopy expression of *arfB* in *E. coli* can suppress the synthetic lethality of *ssrA* and *arfA* deletions [30], [31]. However, endogenous *arfB* does not support deletion of *ssrA* in *S. flexneri* or simultaneous deletion of *ssrA* and *arfA* in *E. coli* [30], suggesting that it is not expressed under culture conditions even when nonstop translation complexes accumulate to lethal levels.

## Materials and Methods

### Bacterial strains and plasmids

All strains were grown at 37°C in lysogeny broth supplemented with 30 µg/ml kanamycin, 20 µg/ml chloramphenicol, or 12 µg/ml tetracycline as appropriate (Table 1). Transformation of plasmids into *S. flexneri* was performed by electroporation [32].

**Table 1:** Strains, plasmids and primers used in this study.

Name	Description	Source or reference
Strains		
*E. coli* K-12 MG1655	Wild-type strain	Gift from S. Ades
*Shigella flexneri* 2a 2457T	Wild-type strain	American Type Culture Collection
*S. flexneri* p*SsrA*	Contains plasmid expressing *ssrA* under control of its native promoter	This study
*S. flexneri* p*SsrA* pKD20	Recipient for Red-mediated replacement of *ssrA*	This study
*S. flexneri* pCA24N-His_6_-ArfA	Contains ASKA plasmid with *arfA*	This study
*E. coli* BD1467	Donor *E. coli* strain used to transduce *zfg-2003::Tn10* into *S. flexneri*	Yale Stock Center
*S. flexneri zfg-2003::Tn10 pSsrA*	Recipient strain for Red-mediated replacement of *ssrA*	This study
*S. flexneri zfg-2003::Tn10 ssrA::kan p*SsrA	Donor strain for preparing P1 lysate for co-transduction experiments	This study
Plasmids		
pJS14	Derivative of pBBR1MCS; chlor^R^	[38]
pSsrA	*ssrA* with its endogenous promoter on pJS14	This study
pKD4	Plasmid template used to generate insert for *ssrA* replacement	[33]
pKD20	Red-recombinase expression plasmid	[33]
pCA24N-His_6_-ArfA	ASKA plasmid expressing His-tagged ArfA under control of an IPTG-inducible promoter	[39]
Primers		
Shi_ssrA_del-F	5′-cgacacaaatgttgccatcccattgcttaatcg aatttgagcgattgtgtaggctggagctgcttc -3′	This study
Shi_ssrA_del-R	5′-tcggatgactctggtaatcaccgatgga gaattttgatgggaattagccatggtcc -3′	This study
ssrAU_BamHI	5′-acgggatccctcttattggctatcacatc-3′	This study
ssrAL_HindIII	5′-cgtcgtaagctttaaaaggttcggatttaa -3′	This study
ssrA_KO_check-F	5′-aattattgaccagttcctcaccgcgcctc-3′	This study
ssrA_KO_check-R	5′-gttggcatcagacttcgcgggacaaattcg -3′	This study

The sequences of *ssrA* genes from *E.coli* and *S. flexneri* are identical. Plasmid p*SsrA* was made by amplifying *ssrA* from *E. coli* K-12 MG1655 using primers ssrAU_BamHI and ssrAL_HindIII, digesting the product with BamHI and HindIII, and ligating the resulting DNA into pJS14 cut with the same enzymes. Red-mediated recombination was performed using the Wanner method [33]. *S. flexneri* cells containing pKD20 and p*SsrA* were transformed with a PCR product made using primers Shi_ssrA_del-F and Shi_ssrA_del-R with plasmid pKD4 as the template. *E. coli* strain BD1467 was used as the donor strain to transduce *zfg-2003::Tn10* mutation into *S.flexneri.* Plasmid pCA24N-His_6_-ArfA was a gift from the Ades lab, and the sequence of *arfA* on the plasmid was verified prior to use.

### P1 transduction

P1 lysates were prepared from *E. coli zfg-2003::Tn10* and *S. flexneri zfg-2003::Tn10 ssrA::kan* p*SsrA* according to published protocols [34]. For transductions, cells of the recipient strain were harvested from 1.5 ml saturated culture and resuspended in 0.75 ml P1 salts solution (10 mM CaCl_2_, 5 mM MgSO_4_). 0.1 ml cell suspension was incubated with 1, 10, or 100 µl P1 lysate for 30 min at 37°C. After incubation, 1 ml lysogeny broth and 0.2 ml 1 M sodium citrate were added and the samples grown 1 h at 37°C with aeration. Cells were harvested by centrifugation, resuspended in 50 µl lysogeny broth and grown on LB plates with the appropriate antibiotic at 37°C. The expected co-transduction frequency was calculated according to the formula [1-(d/L)]^3^ [35].

### PCR to verify gene replacement

Replacement of *ssrA* in *S. flexneri ssrA::kan pSsrA* was verified by colony PCR using primers ssrA_KO_check-F and ssrA_KO_check-R, which flank the *ssrA* gene. As a control, colony PCR using the same primers was also performed on wild-type *S. flexneri*. To verify replacement of *ssrA* in *S. flexneri ssrA::kan pCA24N-His_6_-ArfA*, genomic DNA was prepared from the deletion strain [36], and used as template for PCR amplification using primers ssrA_KO_check-F and ssrA_KO_check-R. As a control, genomic DNA was prepared from wild-type *S. flexneri* and used as a template for PCR amplification using the same primers. The expected product size for wild-type was 681 bp, and for *ssrA::kan* the expected product size was 1724 bp.

### ArfA expression and growth

Expression of ArfA in *S. flexneri ssrA::kan pCA24N-His_6_-ArfA* was examined under three different conditions. Saturated cultures of *S. flexneri ssrA::kan pCA24N-His_6_-ArfA* grown with or without IPTG were diluted 1∶100 into growth medium with 1 mM final concentration of IPTG, or cells were grown without any exposure to the inducing agent. As a negative control, wild-type *S. flexneri* without plasmid was tested. Cultures were grown to OD_600_ = 0.4 at 37°C, and cells were harvested by centrifugation and analyzed by Western blotting.

Growth curves were obtained by diluting saturated cultures of *S. flexneri ssrA::kan* p*CA24N-His_6_-ArfA* 1∶100 in growth medium with or without 1 mM IPTG at 37°C with constant shaking, and sampling cultures every 20 min to measure OD_600_. Points between 80 min and 160 min were fit to the single exponential function OD_600_ = c(e^bt^), where t is time, and the value for b was used as the growth rate.

### Western blotting

Cell pellets were lysed by boiling in SDS sample buffer (63 mM Tris-HCl (pH 6.8), 2% SDS, 10% glycerol, 0.1% 2-mercaptoethanol, 0.0005% bromophenol blue). The samples were resolved on a 15% SDS polyacrylamide gel, blotted to PVDF membrane, and probed with 1∶5000 dilution anti-PentaHis antibody (Qiagen) [37]. Goat anti-mouse antibody (GE Healthcare) was added at 1∶5000 dilution for 1 h at room temperature prior to addition of ECF reagent and imaging with a Typhoon 9410 (GE Healthcare). The relative amounts of ArfA protein were determined by quantifying the bands using InageQuant software (GE Healthcare).
